# Variation in defence strategies in the metal hyperaccumulator plant *Noccaea caerulescens* is indicative of synergies and trade-offs between forms of defence

**DOI:** 10.1098/rsos.172418

**Published:** 2019-01-23

**Authors:** Helen N. Fones, Gail M. Preston, J. Andrew C. Smith

**Affiliations:** 1 Biosciences, University of Exeter, Geoffrey Pope Building, Stocker Road, Exeter EX4 4QD, UK; 2 Department of Plant Sciences, University of Oxford, South Parks Road, Oxford OX1 3RB, UK

**Keywords:** *Noccaea*, hyperaccumulator, *Pseudomonas*, zinc, cell death, glucosinolates

## Abstract

In the metal hyperaccumulator plant *Noccaea caerulescens,* zinc may provide a defence against pathogens. However, zinc accumulation is a variable trait in this species. We hypothesize that this variability affects the outcome of interactions between metal accumulation and the various constitutive and inducible defences that *N. caerulescens* shares with non-accumulator plants. We compare zinc concentrations, glucosinolate concentrations and inducible stress responses, including reactive oxygen species (ROS) and cell death, in four *N. caerulescens* populations, and relate these to the growth of the plant pathogen *Pseudomonas syringae*, its zinc tolerance mutants and *Pseudomonas* pathogens isolated from a natural population of *N. caerulescens.* The populations display strikingly different combinations of defences. Where defences are successful, pathogens are limited primarily by metals, cell death or organic defences; there is evidence of population-dependent trade-offs or synergies between these. In addition, we find evidence that *Pseudomonas* pathogens have the capacity to overcome any of these defences, indicating that the arms race continues. These data indicate that defensive enhancement, joint effects and trade-offs between different forms of defence are all plausible explanations for the variation we observe between populations, with factors including metal availability and metal-tolerant pathogen load probably shaping the response of each population to infection.

## 1. Introduction

Metal hyperaccumulation, defined as uptake and storage of exceptionally high concentrations of a metal in the aerial tissues of a plant [1], is a relatively uncommon trait, documented in around 600 species of vascular plants [2–4]. A substantial number of these are found within the family Brassicaceae, but examples occur throughout the plant kingdom, from the arsenic-hyperaccumulating fern *Pteris vittata* [5] to the montane crucifer *Noccaea caerulescens*, which can accumulate zinc, nickel and cadmium to many times higher than normal physiological concentrations [6,7]. Multiple evolutionary origins have been postulated for this trait [8–11].

Among these, the hypothesis that metal hyperaccumulation evolved because the metals provided an effective defence against herbivores or pathogens has received support in recent years [12–14]. Previously, we have shown that zinc hyperaccumulation by *N. caerulescens* is able to restrict the growth of the common bacterial pathogen *Pseudomonas syringae* pv. maculicola (*Psm*) through direct toxicity of the metal ion itself [13]. We confirmed this by using mutants of *Psm* with altered zinc tolerance, which showed corresponding changes in their ability to grow in high-zinc plants. Further work showed that, in *N. caerulescens*, some of the inducible defences common in non-hyperaccumulating plants have been lost or altered, with salicylic acid signalling and downstream-induced defences uncoupled from the reactive oxygen species (ROS) signalling that normally precedes them [15,16]. We hypothesized that this may be a necessary result of the plants' ability to tolerate exceptionally high metal concentrations, which themselves induce ROS [17–20]. These findings are in line with earlier findings such as those of Plessl *et al.* [21], who discovered a trade-off between zinc tolerance and the expression of defence-related genes.

*Noccaea caerulescens* is a variable species [6,22–24] whose European populations differ in both morphological and physiological features, including metal hyperaccumulation [25–30]. Populations exhibit variable degrees of metal tolerance and of metal accumulation, which are thought to be genetically independent traits [6,31]. These differences may be reflected in altered outcomes of the trade-off between metal accumulation and defence signalling, and thus in differences in the importance of metals in defence.

It has been suggested that variation in metal hyperaccumulation cannot explain all observed differences in palatability to herbivores [32,33], with glucosinolates postulated as the defensive compound of most importance in such interactions. These findings appear incompatible with the idea that the evolution of metal-based defences has led to a trade-off with all organic forms of defence. However, the relationship between metal hyperaccumulation, glucosinolate production and disease or pest susceptibility in *N. caerulescens* and other metal-hyperaccumulating plants is complex and highly dependent upon the *N. caerulescens* population, the hyperaccumulated metal and the availability of that metal, as well as the class of glucosinolates considered [34]. As a result, studies have come to differing conclusions concerning metal accumulation–glucosinolate production trade-offs. Evidence for trade-offs between metal and glucosinolate concentrations was found in *Streptanthus polygaloides* hyperaccumulating nickel [35] and in *N. caerulescens* hyperaccumulating cadmium or zinc [36,37]. There is also evidence that cadmium can suppress glucosinolate production in the non-accumulator brassica *Arabidopsis thaliana* [38]. Contrasting results, in which hyperaccumulation of metals leads to an increase in glucosinolates, have been found in *N. caerulescens* hyperaccumulating nickel [36], in *Noccaea (Thlaspi) praecox* accumulating cadmium [36], and in *Brassica oleracea* exposed to high zinc or cadmium [39]. Additionally, more complex interactions are sometimes seen whereby the same metal might induce certain types of glucosinolates and suppress others, without necessarily affecting the overall glucosinolate concentration [38].

The variability in the reported data suggests that different selection pressures have led to different outcomes in particular hyperaccumulator species or populations. Glucosinolate production in non-hyperaccumulator plants is affected by a number of plant hormones with roles in plant defence against pathogens, pests and wounding, including ethylene, indole acetic acid (IAA), jasmonic acid (JA) and salicylic acid (SA) [35,39–42]. Production of these hormones can also be affected by heavy metal stress [43], as can glucosinolate production [37,39,44]. In addition, there is some evidence that biotic and abiotic stresses can induce increased metal accumulation in certain hyperaccumulators [45], as well as expression of the *HMA4* zinc transporter gene *N. caerulescens* [37]. As a result, plant hormones, metals and glucosinolates form an inter-related network. This might allow fine-tuning of the balance between these three aspects of plant defence, both as a short-term response and also over evolutionary time.

In this work, we investigate the importance of zinc hyperaccumulation and other defences in four populations of *N. caerulescens* known to differ in their zinc tolerance and hyperaccumulation in the field [22,28], as well as in their palatability and vulnerability to herbivory [32,33]. We test the hypotheses that (i) inter-population differences in zinc accumulation are reflected in differences in vulnerability to pathogens or (ii) differences in non-metal-based, inducible and non-inducible defences [16]. To address the first of these hypotheses, we study the growth of *Pseudomonas syringae* pv. maculicola M4 (*Psm*) and zinc tolerance mutants of this pathogen, and of two naturally occurring endophytes of *N. caerulescens* [46] *in planta* in all four populations. To address the second hypothesis, we investigate glucosinolate concentrations and two inducible stress responses previously found to be altered in *N. caerulescens*, when compared to non-hyperaccumulating species—ROS production and cell death [16].

## 2. Material and methods

### 2.1. Plants

Seeds of *Noccaea caerulescens* J. & C. Presl from natural populations in Prayon (Belgium), Ganges (France), Viviez (France) and Wilwerwiltz (Luxembourg) (provided by A.J.M. Baker, C. Lefèbvre and N. Noret) were cultured hydroponically on modified 0.1-strength Hoagland solution [47,48] in a glasshouse in which supplemental sodium-vapour lamps were used for 14 h per day. Temperature was maintained at a minimum of 24°C (day) or 14°C (night). Hoagland solution was supplemented with 0.04, 10, 30 or 300 µM ZnSO_4_, as described [13]. These four populations of *N. caerulescens* were chosen to represent the range of metal hyperaccumulation phenotypes seen in this species. Prayon is a well-characterized accession, with a hyperaccumulation phenotype that might be considered typical for metalliferous *N. caerulescens,* and has been used by us in previous studies [13,16,46]. Viviez and Ganges are both metalliferous accessions with high tolerance to zinc, but Ganges is also remarkable in its tolerance and accumulation of cadmium [6,49,50]. Wilwerwiltz, meanwhile, represents a non-metalliferous ecotype of *N. caerulescens* [6].

*Arabidopsis* *thaliana* (Col-0) plants were grown on John Innes no. 2 compost under the same conditions for six weeks.

### 2.2. Measurement of zinc concentrations *in planta*

Whole-leaf zinc concentrations were measured as described in [13]. Briefly, fresh leaf material was oven-dried, digested in concentrated nitric acid, diluted 10-fold with ultrapure water and filtered. Metal concentrations were measured in an air–acetylene flame by atomic absorption spectrophotometry, using a double-beam optical system with deuterium arc background correction (AAnalyst 100; Perkin-Elmer, UK). Two independent, replicate experiments were performed, in which the zinc concentration in plants of the four populations, each grown on each of the four metal regimes for eight weeks, was measured. For each treatment/population combination, three biological replicates, each consisting of pooled leaf tissue from 5 to 10 plants, were used; for each of these biological replicates, six technical replicates were measured.

### 2.3. Bacteria

Strains were maintained at −80°C in 50% (v/v) glycerol, and unless otherwise stated were grown on KB agar at 28°C. Zinc tolerance mutants of *Psm* were created by transposon mutagenesis as described in [13]. Strain 9A6, in which the insertion disrupts a proline iminopeptidase gene (PSPTO_5164), has increased zinc tolerance compared to wild-type, while 10C1, in which a putative TonB-dependent DNA ligase (PSPTO_2152) is disrupted, has decreased zinc tolerance [13]. Strains SnC10 and SnB11 were isolated from leaves of a naturally occurring population of *N. caerulescens* at Hafna mine, Snowdonia, and are discussed in detail in [46].

### 2.4. *In planta* growth assays

These were carried out as described previously [13]. Briefly, bacteria were suspended in sterile 10 mM MgCl_2_ at approximately 10^6^ cfu ml^−1^ (see, for example, [51]). This suspension was infiltrated into fully expanded *N. caerulescens* or *A. thaliana* leaves through the abaxial surface. Nine leaves on each of six plants were inoculated within each treatment/population combination. Leaf discs were taken from three of the inoculated leaves at 0, 2 or 5 days post-inoculation, homogenized in 10 mM MgCl_2_ and spread onto *Pseudomonas*-selective media. At least three replicate plates were used for each such sample. After incubation at 28°C for 48 h, bacterial colonies were counted.

### 2.5. Cell death staining with trypan blue

*Noccaea caerulescens* plants were treated with 0.04, 10, 30 or 300 µM zinc. Three leaves of each of six plants were infiltrated with *P. syringae* pv. maculicola M4 at 10^7^ cfu ml^−1^ in 10 mM MgCl_2_, with 10 mM MgCl_2_, or left untreated. *Arabidopsis thaliana* plants were inoculated with *Psm* at 10^5^ cfu ml^−1^. After 48 h, leaves were excised and stained overnight in 0.1% (w/v) trypan blue before decolouring in 100% methanol. Decoloured leaves were photographed and images were analysed for percentage area coloured blue using specifically written software as described in [16].

### 2.6. Staining for hydrogen peroxide and superoxide

*Noccaea caerulescens* plants were treated with 0.04, 10, 30 or 300 µM zinc. Three leaves of each of six plants were infiltrated with *P. syringae* pv. maculicola M4 at 10^7^ cfu ml^−1^ in 10 mM MgCl_2_, with 10 mM MgCl_2_, or left untreated. *Arabidopsis thaliana* plants were inoculated with *Psm* at 10^5^ cfu ml^−1^. After 1, 2 or 6 h, leaves were excised and stained overnight in 0.01% (w/v) DAB (3,3-diaminobenzidine, for H_2_O_2_) or NBT (Nitroblue tetrazolium, for O_2_^•−^) solution before decolouring in 100% methanol. Decoloured leaves were photographed and images were analysed for percentage area coloured brown (DAB) or blue (NBT) using specifically written software as described in [16].

### 2.7. Glucosinolate extraction and measurement

Weighed leaves of *N. caerulescens* were lyophilized and ground to a fine powder before extracting in 70% (v/v) aqueous methanol at 70°C for 20 min. Samples were centrifuged and methanol evaporated from the supernatant before resuspension in 1 ml ultrapure water [52,53]. Samples were then incubated with 5 µl thioglucosidase (0.5 µg ml^−1^) from *Sinapis alba* seed (Sigma Aldrich) for 3 h at room temperature. This enzyme cleaves one molecule of glucose from each molecule of glucosinolate [54–56]. Glucose concentrations were measured using the Glucose (GO) Assay Kit (Sigma, UK) according to the manufacturer's specification. Controls were prepared by adding 1% (v/v) glacial acetic acid to the methanol during extraction to inactivate native thioglucosidase and provide a measure of endogenous glucose [57]. These control samples were subsequently treated with 5 µl distilled water in place of thioglucosidase. For each plant type and zinc treatment, three control and five biological replicates were analysed. The experiment was performed three times with independent sets of plants. To validate the method, small quantities (0.25 or 0.5 mg in aqueous solution) of the commercially available glucosinolate, sinigrin, were added to three samples each of ground leaves (0.005 g, Prayon, 10 µM Zn) prior to extraction. These spiked samples were then treated the same as the experimental samples, and concentration estimates of greater than 50 or 100 g of sinigrin per g dried leaf were confirmed, respectively.

### 2.8. Statistical analyses

Datasets were analysed using analysis of variance (ANOVA) with simultaneous Bonferroni comparisons, carried out in Minitab (Minitab Inc., Coventry, UK). In figures, the data points whose means were found to be significantly different in Bonferroni comparisons are marked with different letters. Datasets were tested for homogeneity of variance using the *F*_max_ test [58,59] and transformed where necessary, prior to analysis, to meet this assumption of ANOVA, using either square root or log transformations as appropriate. Further details are given in figure legends. Unless otherwise stated, *α* = 0.05

## 3. Results

### 3.1. All populations accumulate zinc in proportion to zinc treatment applied

**Figure 1.**
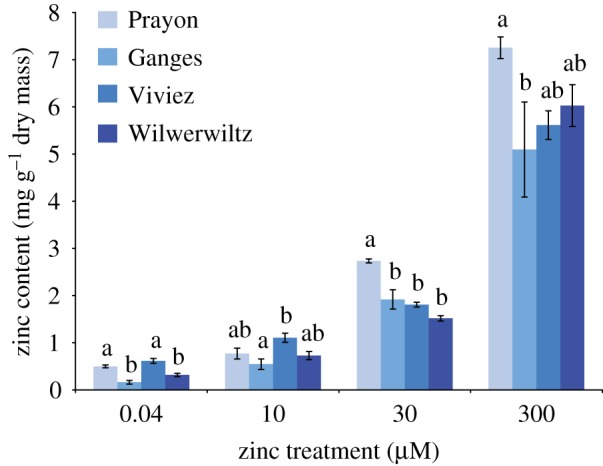
Zinc concentrations in *N. caerulescens* bulked leaf samples from four populations. *Noccaea caerulescens* plants were grown on 0.1× Hoagland solution supplemented with 0.04, 10, 30 or 300 µM Zn for eight weeks. Five to ten plants per treatment were dried, pooled and acid-extracted. Zinc concentrations were measured by atomic absorption spectrophotometry with six technical replicates per sample. Two independent experiments were carried out, of which data presented are means ± s.e. Population is a significant predictor of zinc concentration in each zinc treatment (ANOVA; *p <* 0.0005, 0.004, *<*0.0005 and 0.089). Data are means of two independent experiments and error bars indicate standard error. Means that are not significantly different (Bonferroni simultaneous comparisons) are marked with the same letter; letters apply only within the group compared by each ANOVA, i.e. within zinc treatments.

It has been reported that plants of the different *Noccaea caerulescens* populations in Europe have differing capacities for zinc accumulation [32,33,48]. We therefore measured zinc concentrations in the leaves of plants of the four different populations studied here when grown hydroponically under standardized conditions in modified 0.1× Hoagland solution amended with 0.04, 10, 30 or 300 µM zinc (figure 1). All plants accumulated increased amounts of zinc when grown on the higher zinc treatments. At 30 and 300 µM zinc, Prayon plants accumulated the highest foliar concentrations of zinc (7.4 and 2.7 mg g^−1^ dry weight), whereas plants from Ganges (one of the most metal-tolerant populations of this species) accumulated significantly less zinc than Prayon at these two treatment concentrations (5.1 and 1.9 mg g^−1^ dry weight), showing no significant difference in zinc concentration from the Viviez and Wilwerwiltz plants. In all four populations, treatment with up to 300 µM zinc caused no visible stress or stunting, with plants showing neither visible chlorosis nor anthocyanin production.

### 3.2. ROS responses to *Psm* do not vary between populations, but the cell death response does

Previous work with *N. caerulescens* has shown that, in the Prayon population, pathogen-induced defences are uncoupled from ROS signalling [16]. No ROS response to *Psm* was detected, although some defences normally downstream of ROS, such as salicylic acid production, were present, while others, such as induction of *PR* (pathogenesis-related protein) genes, could not be detected. This was attributed to mechanisms for the suppression of metal-induced ROS or ROS-signalling, as pathogen-independent superoxide production was detected. In line with these previous findings, no pathogen-induced increase in H_2_O_2_ or O_2_^•−^ was detected using 3,3′-diaminobenzidine (DAB) or nitroblue tetrazolium (NBT) staining, respectively, in any population in the current work (electronic supplementary material, figures S1 and S2). The only exception was a significant increase in O_2_^•−^ in response to *Psm* in plants of the Viviez population on 0.04 µM zinc (electronic supplementary material, figure S2). All populations showed increased superoxide levels in uninoculated plants compared to *Arabidopsis thaliana*, confirming that previous results from the Prayon population also apply to the other three *N. caerulescens* populations.

**Figure 2.**
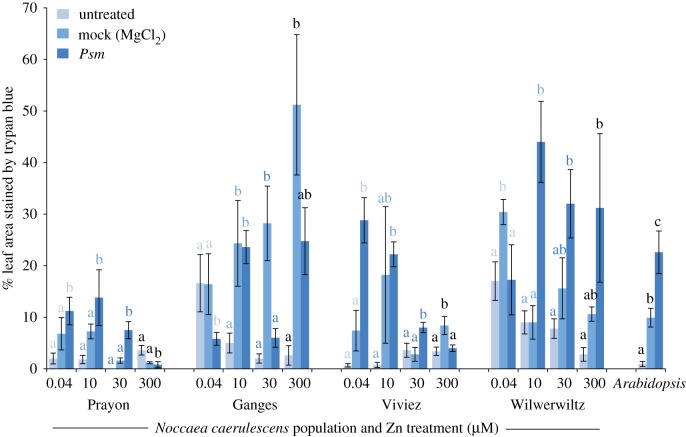
Cell death responses to *Psm* or mock (MgCl_2_) inoculation in *N. caerulescens* plants of the four populations, and in *A. thaliana*. *Noccaea caerulescens* plants were treated with 0.04, 10, 30 or 300 µM zinc. Three leaves of each of six plants were infiltrated with *P. syringae* pv. maculicola M4 at 10^7^ cfu ml^−1^ in 10 mM MgCl_2_, with 10 mM MgCl_2_, or left untreated. After 24 h, leaves were excised and stained overnight in 0.1% (w/v) trypan blue before bleaching and image analysis. ANOVAs were used to test for significant effects of inoculum within each population/zinc combination. Where inoculations significantly (*α* = 0.05) affect trypan blue staining, Bonferroni simultaneous comparisons were carried out and significantly different means are indicated with different letters. Letters only apply within each zinc treatment; for clarity, letters pertaining to different zinc treatments within each population are coloured differently. Results shown here represent the mean of two independent experiments. Error bars indicate s.e.

Cell death responses, however, were found to vary between populations (figure 2). Plants of the Prayon population showed little or no trypan blue staining if uninoculated, and no significant response to mock inoculation, with small but significant levels of cell death in response to *Psm*, at low zinc concentrations, and no cell death in response to *Psm* at 300 µM zinc. The Viviez population showed the same pattern of cell death responses, although these responses were stronger (up to 30% of leaf area stained by trypan blue, compared to 12% in Prayon plants) and their decrease at higher zinc concentrations was more marked. By contrast, both Ganges and Wilwerwiltz plants showed high levels of cell death, even in the absence of inoculation, if grown on low zinc. This is suggestive of a highly sensitive or spontaneous cell-death response, which is suppressed when the plants are grown on high zinc. In response to both mock and *Psm* inoculation, these plants showed extensive cell death, leading to 45–50% of the leaf area being stained by trypan blue. Unlike the cell death response in Prayon and Viviez plants, this response was not suppressed by zinc. Additionally, at 0.04 µM zinc, *Psm* inoculation led to reduced cell death compared to mock inoculation in both of these populations.

### 3.3. The pattern of glucosinolate production in response to zinc is highly population dependent

**Figure 3.**
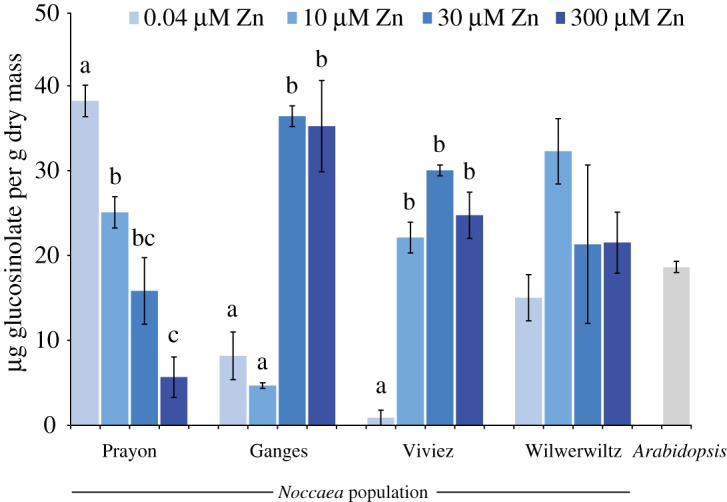
Glucosinolate concentrations in *N. caerulescens* plants of the four populations, and in *A. thaliana.* Five biological replicates were used for each population/zinc treatment combination. ANOVAs were used to test for significant effects of zinc on glucosinolate concentration within each population. Where zinc significantly (*α* = 0.05) affects glucosinolate concentrations, Bonferroni simultaneous comparisons were carried out and significantly different means are indicated with different letters. Results shown here represent the mean of two independent experiments. Error bars indicate s.e.

Glucosinolate production has been postulated to be important in explaining the susceptibility of *N. caerulescens* plants from different populations to herbivory. We therefore measured glucosinolate concentrations in the plants used in this study (figure 3). In plants of the Prayon population, there appears to be a trade-off between zinc accumulation and glucosinolate production, with plants grown on increasing zinc concentrations containing progressively less glucosinolates. Ganges and Viviez populations showed the opposite pattern, with increased zinc-inducing glucosinolates, while Wilwerwiltz plants showed no significant changes in glucosinolate concentrations in response to zinc. To determine whether these inter-population differences might be important in explaining pathogen growth *in planta*, we tested the ability of five pathogen strains to grow on media supplemented with the commercially available glucosinolate sinigrin. None of the bacteria were sensitive to sinigrin alone, but when the enzyme myrosinase was also added to the growth medium, leading to the release of sinigrin breakdown products, all were susceptible (electronic supplementary material, figure S3). Half-maximal inhibition of growth for all three *Psm* strains was achieved at around 2 mM sinigrin in the presence of myrosinase. Strain SnC10 had a similar sensitivity, while half-maximal inhibition of SnB11 occurred at around 1 mM sinigrin in the presence of myrosinase (electronic supplementary material, figure S3).

### 3.4. Zinc hyperaccumulation cannot explain all observed failures of pathogen growth

**Figure 4.**
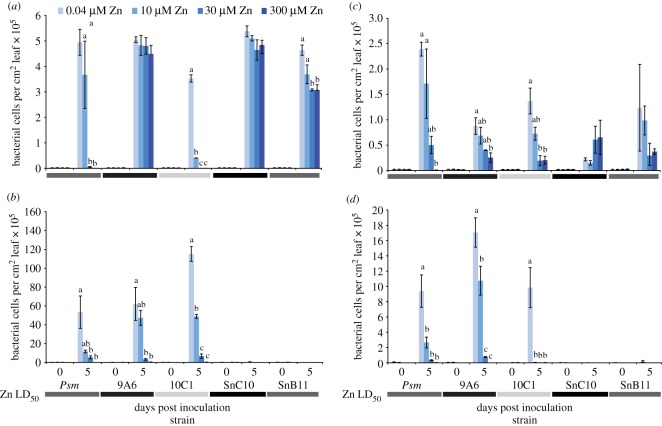
Growth of *Pseudomonas syringae* pv. maculicola, zinc tolerance mutants and naturally occurring endophytes isolated from *N. caerulescens* at Hafna mine, UK, in plants of the four populations. Growth over five days is shown in plants of the (*a*) Prayon, (*b*) Ganges, (*c*) Viviez and (*d*) Wilwerwiltz populations. *Noccaea caerulescens* plants were treated with 0.04–300 µM zinc. Nine leaves of each of six plants were infiltrated with: *P. syringae* pv. maculicola M4 (Zn LD_50_ 7.3 mM); the increased zinc tolerance mutant of *Psm*, 9A6 (Zn LD_50_ 10.0 mM); the reduced zinc tolerance mutant of *Psm*, 10C1 (Zn LD_50_ 2.3 mM); the naturally occurring *N. caerulescens* endophyte SnC10 (Zn LD_50_ 11.8 mM) or the naturally occurring *N. caerulescens* endophyte SnB11 (Zn LD_50_ 7.1 mM), suspended in 10 mM MgCl_2_ at 10^6^ cfu ml^−1^. Heat maps beneath *x*-axes indicate Zn LD_50_ for each strain, with lighter colour representing lower zinc tolerance. Leaves were sampled at 0 and 5 days after inoculation. Six samples were taken per time point and treatment, each sample consisting of three leaves pooled from one plant. The experiment was repeated twice with similar results. Values are means ± s.e. (*n* = 6). Growth of each bacterial strain at different zinc concentrations was compared using ANOVA with Bonferroni simultaneous comparisons; data were square-root transformed for analysis to meet the assumptions of the method. Significant differences are indicated by different letters shown above the graphs; letters apply only within the group compared by each ANOVA, i.e. within bacterial strains. No letters are shown where ANOVAs indicated no significant differences in growth at different zinc concentrations.

To determine whether zinc is an important antimicrobial defence in all populations of *N. caerulescens*, as previously observed in Prayon plants, we tested the ability of wild-type *Psm* and previously described *Psm* zinc tolerance mutants to grow *in planta* in the Ganges, Viviez and Wilwerwiltz populations as well as Prayon (figure 4). In addition, we tested the growth of two *Pseudomonas* strains isolated from a natural population of *N. caerulescens* occurring on mine spoil enriched in zinc [13]. These strains, SnB11 and SnC10, have zinc tolerances similar to wild-type *Psm* and to the increased zinc tolerance *Psm* mutant, 9A6, respectively. In the Prayon population (figure 4*a*), strains grew as previously reported, with the zinc tolerance of the bacterial strains clearly determining their ability to grow in plants cultivated on high-zinc treatments (30 or 300 µM Zn).

This correlation between bacterial zinc tolerance and growth *in planta* was not seen in the other three populations. Zinc accumulation protected *N. caerulescens* plants from *Psm* in Ganges (figure 4*b*), Viviez (figure 4*c*) and Wilwerwiltz (figure 4*d*) plants. However, the *Psm* mutant, 9A6, which has increased zinc tolerance compared to wild type, did not outgrow wild-type *Psm* in high-zinc plants of any of the other three populations. This indicates that, unlike Prayon plants, the protection conferred by zinc accumulation to plants from Ganges, Viviez or Wilwerwiltz is not solely dependent on direct zinc toxicity, because it cannot be overcome by high bacterial zinc tolerance. In Viviez plants (figure 4*c*), growth of all *Psm* strains in low-zinc plants was only approximately half that seen in Prayon (figure 4*a*). Further reduction in bacterial growth was then seen in all cases as the zinc treatment of the plants increased. The two naturally occurring *Pseudomonas* strains, SnB11 and SnC10, both showed low growth in Viviez plants (figure 4*c*) that was not significantly affected by plant zinc content.

The Ganges (figure 4*b*) and Wilwerwiltz (figure 4*d*) populations gave similar results. In both populations, *Psm* and its mutants grew extremely well in low-zinc plants, reaching an order of magnitude higher population density than in low-zinc Prayon plants. However, as in Viviez (figure 4*c*), *Psm* strains showed growth inhibition in high-zinc plants that was not affected by the zinc tolerances of strains. Most strikingly of all, both SnC10 and SnB11 showed extreme inhibition of growth in these two populations, regardless of plant zinc treatment.

No correlation was detected between the level of glucosinolates in plants and inhibition of bacterial growth, even when the presence or absence of cell death was taken into account (multiple regression analyses, within populations: Prayon, P = 0.05, *R*^2^ = 0.20; Ganges, P = 0.07, *R*^2^ = 0.16; Viviez, 0.23, *R*^2^ = 0.08; Wilwerwiltz, P = 0.09, *R*^2^ = 0.15; across populations, all: P = 0.24, *R*^2^ = 0.25; across populations, including only treatments with trypan blue staining % responses greater than those seen in *Arabidopsis* controls: mock inoculation, 10% or *Psm,* 20%): P = 0.15, *R*^2^ = 0.58).

## 4. Discussion

Zinc may play a role in plant defence through direct toxicity of foliar metal to pathogens [60]. Earlier work, conducted in the Prayon population of *N. caerulescens*, has shown that zinc can directly restrict pathogen growth, with *Psm* strains with altered zinc tolerance behaving as expected under the direct toxicity hypothesis [13]. Under the hypothesis that a trade-off exists between metal hyperaccumulation and other forms of plant defence [16,19], both constitutive and inducible changes in defence might be expected in *N. caerulescens,* compared to related non-accumulator plants such as *Arabidopsis thaliana*. Both of these possibilities are exemplified by the Prayon population, in which metal hyperaccumulation occurs in conjunction with the constitutive loss of ROS-based defences [16], and where high metal availability lessens the inducible glucosinolate and cell death responses. It may not be coincidental that these trade-offs are most evident in the Prayon population, which accumulates significantly high concentrations of foliar zinc than the other populations in this work.

The clear relationship between bacterial zinc tolerance and bacterial growth in Prayon plants can be readily explained by the reliance of these plants upon zinc for defence. It is possible that metal accumulation compensates for deficiencies in other defences such as glucosinolates or cell death. Termed ‘metal therapy' by Poschenrieder *et al.* [60], this idea is compatible with Boyd's proposal [12,61] of ‘defensive enhancement' as a possible mechanism for the transition from metal accumulation to the more extreme levels of foliar metal accumulation seen in hyperaccumulator plants. However, both the cell death response and glucosinolate production remain possible in Prayon plants, and are deployed when zinc is not available.

**Figure 5.**
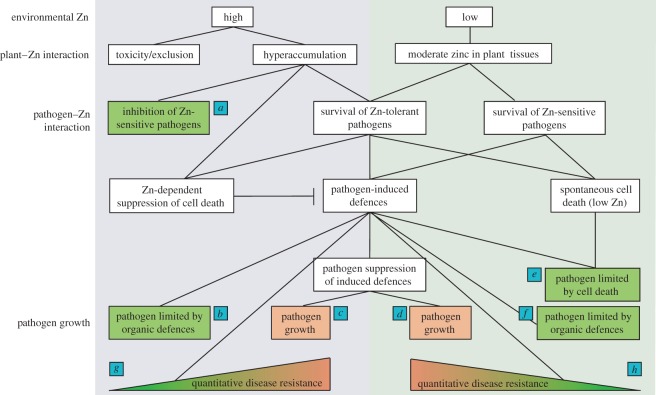
Model showing the various interactions between zinc accumulation, organic defences, cell death defences, pathogen suppression of defences and the various possible outcomes. Depending on the soil on which *N. caerulescens* grows, the plants may contain moderate (right-hand panel) to high (left-hand panel) levels of zinc in their tissues. If the plants have hyperaccumulated zinc and are attacked by zinc-sensitive pathogens (*a*), the pathogen will be suppressed by zinc-dependent defences (seen in Prayon versus *Psm*). Zinc-tolerant pathogens, however, will survive. *Noccaea caerulescens* may then rely upon organic defences to limit pathogen growth (*b*), although these may also act synergistically with cell death defences (e.g. Ganges and Wilwerwiltz). If the zinc-tolerant pathogen can suppress pathogen-induced defences, it will be able to grow (*c*). Where zinc is unavailable, the plants must rely on other defences. Again, if the pathogen can suppress these, it will grow (*d*). Otherwise it may be limited by cell death (*e*; Ganges and Wilwerwiltz) or organic defences (*f*; Wilwerwiltz). In addition, it is possible for the various defences present to result in partial inhibition of the pathogen, known as quantitative disease resistance. This appears to be the case in the Viviez population, both when zinc is available (*g*) and when it is limited (*h*).

In the other *N. caerulescens* populations, the current balance between metal-based defences, glucosinolates and other inducible defences is different. This intraspecific defence variation reflects that seen in the importance of various carbon-based defences within species of subtropical tree [62], suggesting that multiple defensive strategies within one plant species are not uncommon. In figure 5, we attempt to disentangle the various forms of defence for which we have found evidence in the four *N. caerulescens* populations studied here. Prayon plants are represented in case *a*, in which zinc inhibits zinc-sensitive pathogens, preventing disease; however, if pathogens are not zinc sensitive (figure 5*c*), or if insufficient zinc is available for defence (figure 5*d*), the result is pathogen growth and disease. Prayon plants, relying on zinc for defence, are vulnerable in both of these situations.

The Viviez population, like Prayon, shows an apparent defensive trade-off between zinc and pathogen-induced cell death (figure 2). However, in this population, zinc and glucosinolate concentrations are positively correlated (figure 3). Stimulation of glucosinolate production by high zinc may be an example of ‘metal fortification' [60,63], where metals play an indirect role in the protection of the plant from disease by induction of other defences. In the Viviez plants, induction of glucosinolates by zinc may explain the comparatively poor growth of highly zinc-tolerant strains 9A6 and SnC10 in the high-zinc plants of this population, due to glucosinolate release and breakdown during the necrotrophic phase of infection by *Psm*. At low zinc, however, the cell death response to pathogen inoculation remains strong in Viviez plants, and is around three times more extensive than that seen in Prayon. This may explain why all strains failed to thrive in Viviez plants at low zinc, despite the low glucosinolate concentrations seen at low zinc in these plants. In comparison to their growth in low-zinc Prayon plants, all strains were reduced to around 50% population levels in Viviez (figure 4). Thus, Viviez plants display quantitative disease resistance against *Psm* (figure 5*g*) and the naturally occurring *Pseudomonas* strains tested here (figure 5*h*).

‘Metal fortification' can also form an example of what Boyd [12,61] has called ‘joint effects'—whereby synergy between metals and other defences makes metal hypercumulation for defence disproportionally beneficial. The results shown here with the Ganges and Wilwerwiltz ecotypes may represent the result of selection for such joint effects. Both populations show high glucosinolate concentrations, particularly at high zinc, and a cell death response that is positively correlated with zinc accumulation. In these plants at high zinc, cell death may increase the release of both vacuolar zinc and glucosinolate breakdown products, explaining the poor growth of all *Psm* strains, regardless of their zinc tolerance (figure 5*b*). Although it must be remembered that the assay used in this work does not distinguish between different types of glucosinolate, we demonstrate that *Psm* is sensitive to inhibition by allyl isothiocyanate, the product of sinigrin hydrolysis, as previously reported [63]. This supports the idea that release of glucosinolates through cell death contributes to inhibition of *Psm* growth. Sinigrin is known to be produced by *N. caerulescens,* albeit in relatively low quantities compared to sinalbin, which is less toxic to *Psm* [64,65]. However, certain accessions of *N. caerulescens*, including Ganges, produce glucomoringin as a major glucosinolate, the isothiocyanate of which has been reported to have potent anti-microbial activity [65]. De Graaf *et al.* [65] found that the Prayon population was one of only three accessions studied in which sinalbin, rather than glucomoringin, was the main glucosinolate present. These three populations were linked not by metal accumulation phenotype or soil type, but only by geographical closeness, and it is proposed that the dominance of sinalbin reflects a regional genetic difference shared by these populations [65]. Although Viviez and Wilwerwiltz plants were not included in the study, they are not from the same geographical region as Prayon, and so are likely to more closely resemble Ganges plants, in which glucomoringin dominates. If it is true that Prayon plants are distinct among the four populations in the current work in lacking glucomoringin, this could explain why they are also the most dependent on metal-based defences. Thus it would be interesting to study the glucosinolate profiles of all four populations to confirm the hypothesis that Prayon is an outlier in lacking glucomoringin, and to determine the effect of glucomoringin-derived isothiocyanates on *Psm*.

These findings in the Ganges and Wilwerwiltz populations suggest that extreme caution is needed when extrapolating information concerning hyperaccumulator defence to populations beyond those it originates from. For example, Noret *et al.* [33] found that glucosinolates are more important for defence in *N. caerulescens* than zinc, but did not use Ganges or Wilwerwiltz in their work; were their results to be applied to these populations, they may be confounded by the interrelation of defensive factors that we have uncovered in those plants.

At low zinc, however, the Ganges and Wilwerwiltz plants are, like the Prayon plants, susceptible to *Psm.* This could be because they lack the defences ‘fortified' by zinc—glucosinolates and pathogen-induced cell death—as well as zinc itself. Notably, *Psm* is much more successful in Ganges and Wilwerwiltz plants grown at 0.04 µM zinc than that in Prayon plants grown at this zinc concentration. This may be in part because Prayon plants do not rely upon zinc to promote glucosinolate production.

Strikingly, both Ganges and Wilwerwiltz plants displayed high levels of resistance to both SnB11 and SnC10, which showed greatly inhibited growth in these plants at all zinc concentrations. This may indicate an inability of these strains to withstand the combination of cell death, glucosinolates and zinc-based defences that these plants deploy against biotrophic and hemibiotrophic pathogens. This defensive synergy may not be available to the Hafna mine plants from which these bacteria were isolated [13], perhaps due to a zinc–glucosinolate or zinc–cell death trade-off, as seen in Prayon plants. However, zinc and zinc-induced defences cannot explain the poor growth of these two strains in low-zinc Ganges and Wilwerwiltz plants. Both of these populations display a spontaneous cell-death phenotype at low zinc, which does not require the presence of a pathogen. This spontaneous cell death, in combination with glucosinolates, if present (Wilwerwiltz), may prevent the growth of SnB11 and SnC10 (figure 5*e*,*f*). Alternatively, SnB11 and SnC10 may secrete effectors that trigger defence-associated cell death in Ganges and Wilwerwiltz plants, a process known as effector-triggered immunity (ETI) [66–68]. *Psm*, which can grow in these plants, may lack effectors that trigger ETI, or may secrete effectors capable of suppressing ETI (figure 5*c*,*d*). In this case, it must be assumed that the Hafna mine plants in which SnB11 and SnC10 were originally found lack the receptors required to trigger ETI in response to the effector(s) responsible.

## 5. Conclusion

Previously, we showed that ROS-based signalling is uncoupled from defence responses in Prayon *N. caerulescens.* This may prevent metal-induced ROS from causing spurious defence induction [16]. However, it is clear that *N. caerulescens* plants remain at risk from specifically adapted, zinc-tolerant pathogens capable of suppressing cell death. Such pathogens might be expected to arise if local adaptation to a high-metal environment is permitted by the existence of long-standing populations of metal hyperaccumulator hosts in metal-rich environments [13,14]. The current work indicates that cell death remains important for defence against such pathogens in at least some populations of *N. caerulescens* under some zinc regimes.

Various modes of defence, including cell death, glucosinolate production and metal toxicity, are available to *N. caerulescens* plants when attacked by biotrophic bacterial pathogens. Trade-offs may occur between these defences, or they may act synergistically to protect the plant. The precise strategy used appears to depend both on factors which vary between populations and ecotypes of *N. caerulescens,* and on the availability of metal for hyperaccumulation.

## Supplementary Material

Figure S1

## Supplementary Material

Figure S2

## Supplementary Material

Figure S3

## Data accessibility

The data associated with this study can be found in the Dryad Digital Repository: http://dx.doi.org/10.5061/dryad.7tv06 [69].
