# Supplementary figures and images for: Variation in defence strategies in the metal hyperaccumulator plant Noccaea caerulescens is indicative of synergies and trade-offs between forms of defence
Source: R Soc Open Sci. 2019 Jan 23;6(1):172418. doi: 10.1098/rsos.172418 (PMC6366173; doi:10.1098/rsos.172418)

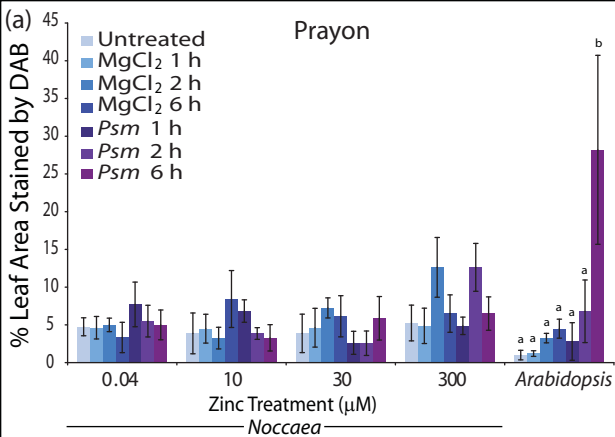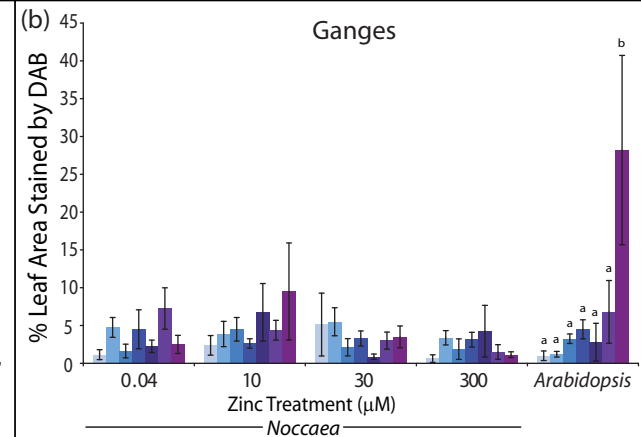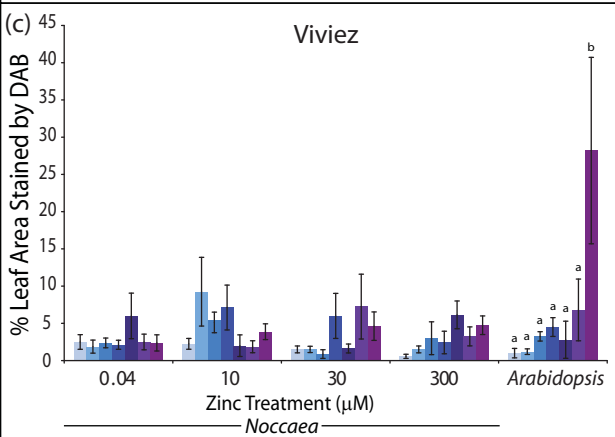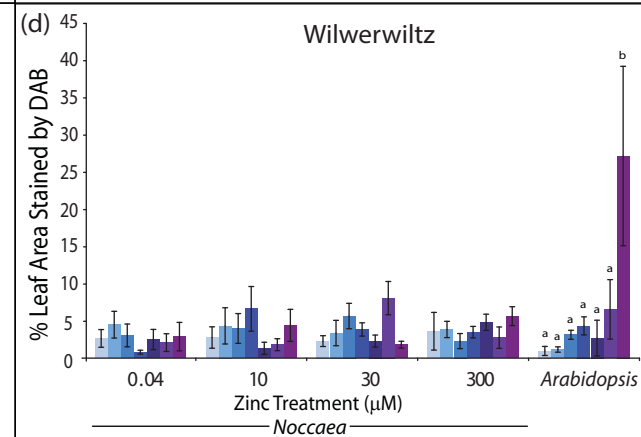

Supplement: Figure S1 [file rsos172418supp1.pdf]

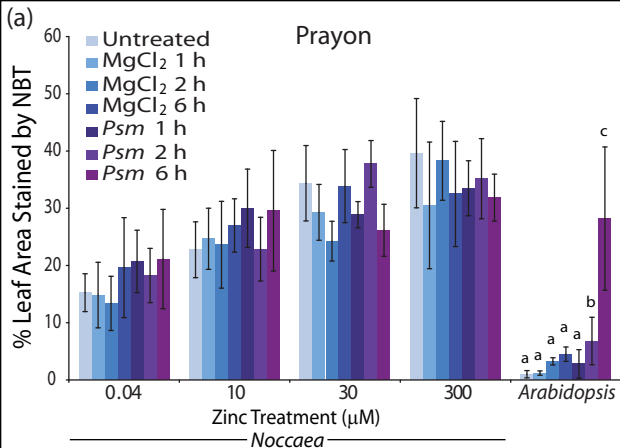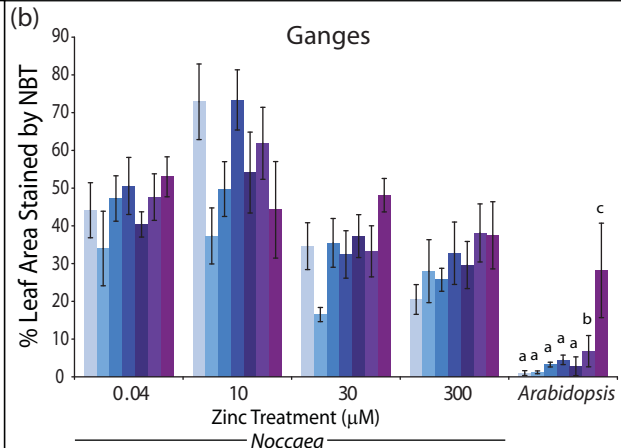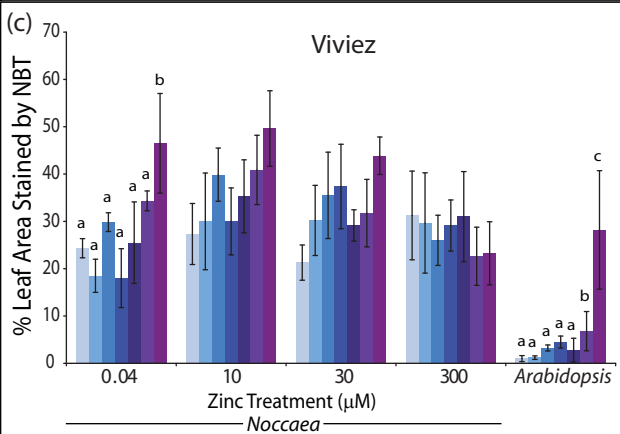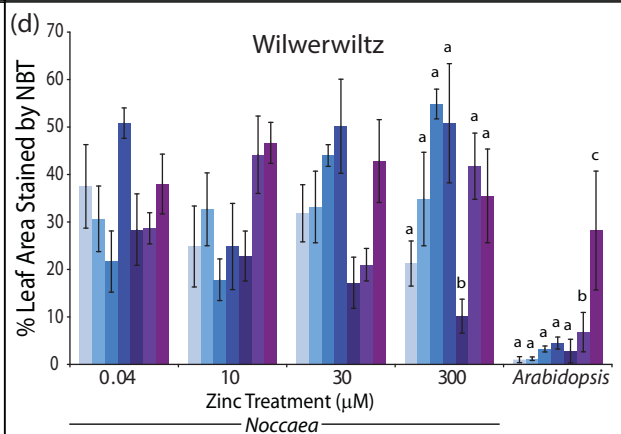

Supplement: Figure S2 [file rsos172418supp2.pdf]

Wildtype Psm

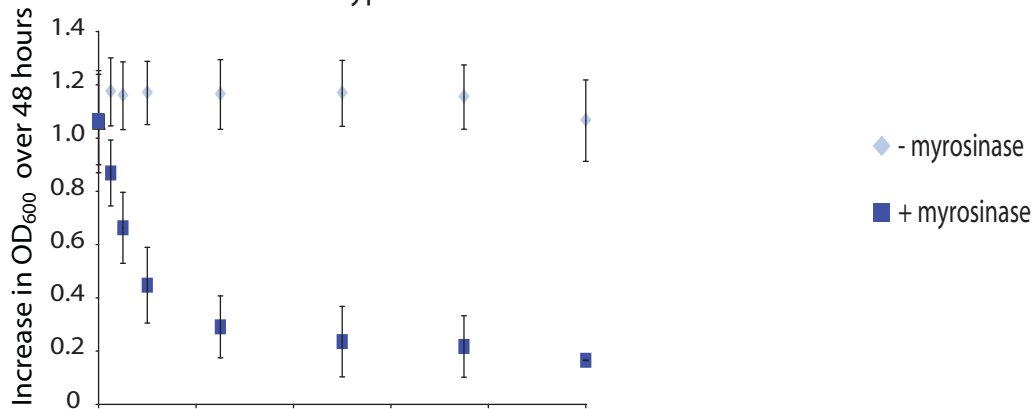

9A6

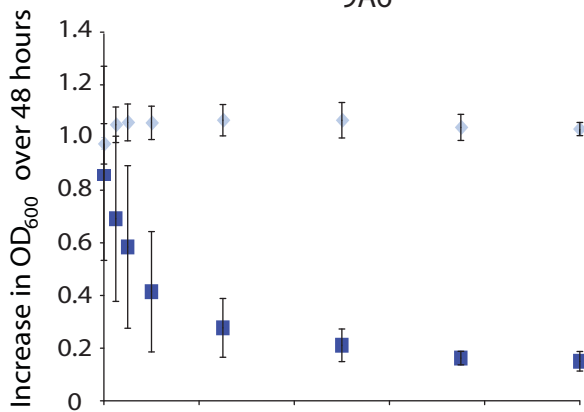

10C1

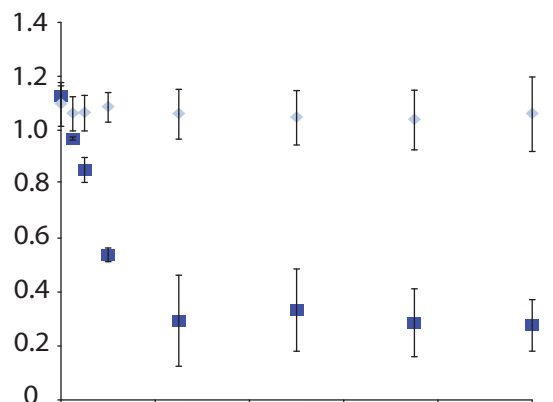

SnC10

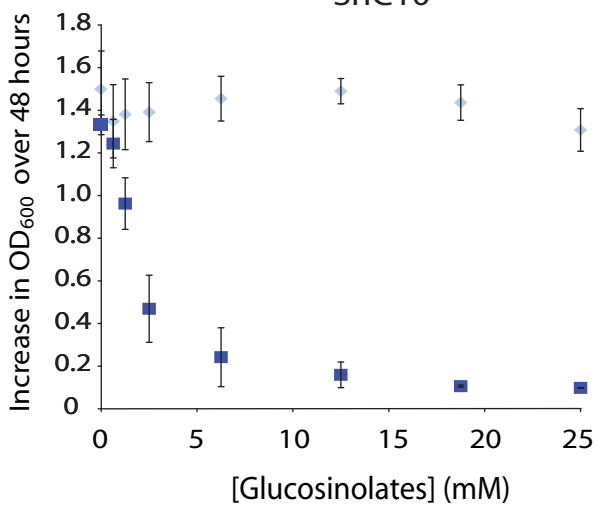

SnB11

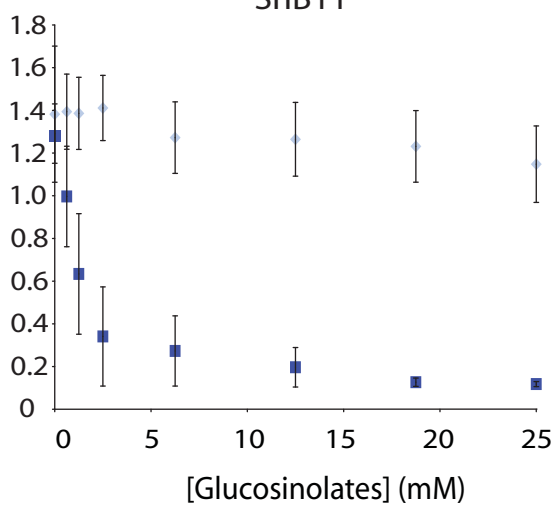

Supplement: Figure S3 [file rsos172418supp3.pdf]
